# Hypertension in Bangladesh: women surpass men at younger ages than worldwide

**DOI:** 10.7189/jogh.15.04138

**Published:** 2025-06-20

**Authors:** Afrin Iqbal, Md Mahabubur Rahman, Mamun Ibn Bashar, Ambar Ahmed, Shusmita Khan, Nahin Ahmed, Mohammad Mehedi Hasan, M Moinuddin Haider

**Affiliations:** 1 Maternal and Child Health Division, icddr,b, Dhaka, Bangladesh; 2 Health Systems and Population Studies Division, icddr,b, Dhaka, Bangladesh; 3 Data for Impact, University of North Carolina at Chapel Hill, Chapel Hill, North Carolina, USA

## Abstract

**Background:**

Worldwide, hypertension is more prevalent among men aged 30 and older, but a national survey from Bangladesh shows a different trend. This paper explores the differences in hypertension by sex and age among the Bangladeshi adult population throughout their life span.

**Methods:**

We conducted the secondary analysis using data from the Bangladesh Demographic and Health Survey (BDHS) 2017–2018. This nationally representative survey involved a two-stage stratified sample of 675 households in urban (250) and rural (425) enumeration areas (EAs). The analytical sample comprised 12 476 individuals aged 18 or older (6955 women and 5521 men). We performed univariate and bivariate tests to investigate variations in hypertension across sex, age, and other factors. We applied multivariable logistic regression models and logit-based marginal probabilities to explore age and sex differences as well as their interactions in hypertension while estimating the marginal effects of sex for each age group to assess the significance of sex differentials in hypertension throughout the life course.

**Results:**

Our study revealed a noteworthy trend: Women have a hypertension prevalence that is five percentage points lower than that of men in their early twenties. However, this trend reverses in their early thirties, with women exhibiting a five-percentage point higher prevalence than men. After adjusting for confounders and age-sex interactions in women, the odds of hypertension were significantly higher (adjusted odds ratio (AOR) = 3.6; 95% confidence interval (CI) = 1.9–6.6) in the 35–39-year-old age group, which may contribute to a combined burden of chronic and reproductive morbidity.

**Conclusions:**

With rising hypertension and stagnant maternal health in Bangladesh, women of reproductive age face higher risks of chronic and reproductive complications. The complex health system in Bangladesh, which starts at the community level, primarily focuses on maternal and child health; however, some recent initiatives show a shift in focus to address significant non-communicable diseases (NCDs) through public health facilities. Considering that more women seek health care from public facilities, recognising common factors contributing to early hypertension in Bangladeshi women enables targeted interventions, model testing, and strategic adjustments to the primary health care-focused national non-communicable disease management pathway.

Hypertension is among the primary preventable risk factors for cardiovascular diseases and accounts for 8.5 million deaths from cardiovascular and cerebrovascular diseases worldwide [1]. A recent publication in The Lancet revealed that the number of hypertensive individuals aged 30 to 79 years worldwide doubled in 2019 compared to 1990, especially in low- and middle-income countries (LMICs) [1,2]. South Asian countries have been the most affected in recent years, with a sharp rise in hypertension leading to a shift in disease burden, where 50% arises from non-communicable diseases (NCDs). A systematic review of studies published between 1969 and 2011 indicated a significant upward trend in the prevalence of hypertension in India [3]. The disease burden attributed to hypertension in India also has risen from 21 million to 31 million between 1990 and 2016 [4]. The prevalence of hypertension among individuals aged 15 to 69 in Nepal rose from 21.5 to 26% between 2008 and 2013 [5]. The prevalence of hypertension, much like in neighboring countries such as Nepal (24%) and Sri Lanka (26%), is becoming more common in Bangladesh as well; in 2011, 22% of adults aged 35 and older had elevated blood pressure (BP), which rose to 35% by 2017 [6-8].

Although certain behavioural factors, such as a sedentary lifestyle, unhealthy diet, high body mass index (BMI), and smoking prevalence, contribute to hypertension in both sexes [9–11], there are non-modifiable factors such as age and sex [8]. Hypertension increases as people age, regardless of sex or ethnicity. However, the global rise in hypertension prevalence with age follows the same pattern in both men and women [8]. Sex differences in hypertension, evident from the literature, show that in most countries, men tend to have a higher prevalence of hypertension than women [8,12,13]. Furthermore, men have a higher rate of hypertension than women of the same age until they reach 65 years old [14]. Some studies indicate that during the menopausal years, which range from ages 45 to 55, women tend to shift from the advantageous status of being normotensive to the disadvantageous state of being hypertensive [14,15]. A similar pattern is observed in our neighboring country, India, where men are more hypertensive than women until they reach 55 years of age; after that point, women surpass men [16].

The roles of sex hormones in the development of hypertension in both men and women, as well as the effects of aging on the cardiovascular system and its connection to other lifestyle-related risk factors, are intricate [17]. There are gender differences in the prevalence of hypertension due to certain biological factors that protect women during adolescence and adulthood. These include sex hormones, chromosomal differences, and other biological sex differences [18,19]. Sex hormones like estrogen and testosterone influence blood pressure regulation. While estrogen is known to help protect women against hypertension, testosterone may contribute to a pro-hypertensive effect in men [18,19]. However, these protective factors decline as women reach menopause, leading to higher hypertension rates in post-menopausal women [11]. These sex hormone-related factors can contribute to the development of hypertension in both men and women, although the impact may differ for each gender [18,19]. Globally, among adults, research indicates that more women are obese while a larger number of men are overweight and smoke. Additionally, men tend to be more physically active than women [20,21]. These behavioural differences, along with the loss of protection from sex hormones in women, may contribute to the gender gap in hypertension observed in various studies.

When considering the sex differences in Bangladeshi adult men and women, the prevalence of hypertension does not align with the typical global pattern in Bangladesh. A higher prevalence is observed among women than men [7,8]. The increasing prevalence of hypertension among women in Bangladesh is a significant concern for the already strained women’s health sector. Hypertension in women of reproductive age (WRA) raises the risk of complications during pregnancy, related maternal morbidities, and mortalities, which can lead to adverse fetal health outcomes [22,23]. Hypertension is a significant risk factor for cardiovascular issues in women throughout their lives [24]. Although more women than men are seeking care and are aware of their hypertension, women in Bangladesh suffer more from hypertension and its related complications, which is counterintuitive [7].

Sex differentials in lifestyle-related risk factors for hypertension vary significantly between men and women, with several factors potentially contributing to this variation. Previous studies on hypertension in Bangladesh primarily concentrated on sociodemographic differences in prevalence, without further exploration of sex differences [6,25]. Research examining trends in hypertension prevalence among men and women in Bangladesh throughout their life course and attempting to create a comparative picture is limited.

Women participate in high labor-intensive work and physical activity less frequently than men [26]. The prevalence of overweight or obesity among women aged 20 to 39 years was 33%, which is 15 percentage points higher than that of their male counterparts [21,27]. Exposure to smoke from solid cooking fuels is linked to a higher risk of hypertension among women [25,28]. Furthermore, combined oral contraceptive (COC) pills are the most commonly used form of modern contraception among married women living in Bangladesh [7]. The use of oral contraceptives and menopause has also been associated with a higher likelihood of women developing hypertension [29]. While one in four COC users in Bangladesh is hypertensive, only two out of five COC users in Bangladesh with elevated blood pressure are aware of their health condition. Exposure to some of these least explored risk factors may vary throughout life and lead to an age difference in the association between gender and hypertension.

A proper understanding of sex differentials by age in hypertension among Bangladeshi adults requires a life course approach. Therefore, this study aims to investigate sex differentials in hypertension across the life course of Bangladeshi adults using data from the Bangladesh Demographic and Health Survey (BDHS) 2017–2018.

This study aims to explore the sex differentials with age in hypertension across the life course among Bangladeshi adults.

## METHODS

### Study design, data, and participants

Data for this study come from the 2017–2018 BDHS, a nationally representative cross-sectional survey based on a two-stage stratified cluster sample of households. In the first stage, 675 enumeration areas (EAs) were selected with probability proportional to EA size (approximately 120 households), consisting of 250 EAs in urban areas and 425 in rural areas. In the second stage, an average of 30 households were systematically selected from each EA. The survey was conducted on a representative sample of 20 250 households and aimed to provide reliable estimates for key health indicators at the national level and in each of the country’s eight divisions.

A subsample of one-quarter of households was selected to measure all household members aged 18 years and older, including individuals, for biomarker and anthropometric measurements (weight, height, blood pressure, and fasting blood glucose). Out of the representative households, 99% of interviews were successfully conducted [7]. Approximately 90% had their blood pressure (BP) measured (14 704). During the BP measurement, it was ensured that all respondents were weighed according to a standard operating procedure. The data collectors underwent rigorous training before data collection to ensure the highest quality data according to the survey protocol. The respondents' BP was measured in their homes, in a relaxed atmosphere while maintaining adequate privacy, and the measurement conditions remained consistent for all respondents.

**Figure 1:**
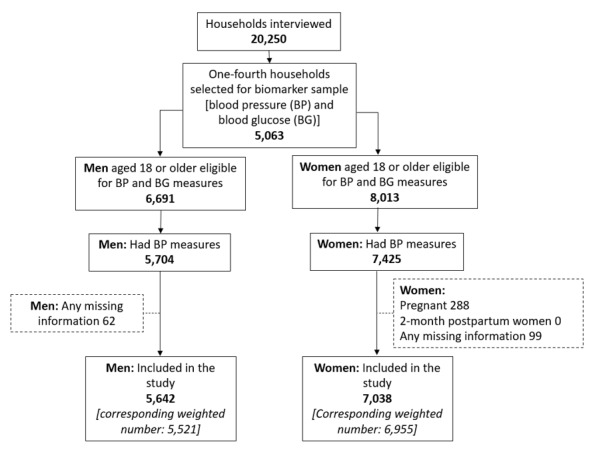
Data flow diagram.

Adequate probes were ensured for self-reported data from the respondents. Furthermore, respondents who did not consent to both the biomarker and anthropometric measurements, as well as pregnant women, women who were two months or less postpartum, and cases with any missing observations were excluded. The reasons for excluding pregnant and postpartum women are to avoid diluting our findings regarding women with normal hypertension *vs*. those with pregnancy-related hypertension, which may resolve naturally in the later postpartum period. Additionally, any missing observations would pose a challenge in exploring the outcome variables in relation to the independent variables. The final analytical sample for this paper comprised 12 476 individuals – 6955 women and 5521 men (**Figure 1**). Please refer to the BDHS 2017–2018 report for more detailed methods.

### Outcome variable

The primary outcome of interest in this study was the hypertension status among adults. LIFE SOURCE® UA-767 Plus BP monitors were utilised to measure BP. Blood pressure was recorded three times by trained technicians, with approximately 10-minute intervals between each measurement, adhering to the World Health Organization (WHO) guidelines for BP measurement. The average of the last two readings was used to classify respondents as hypertensive. The BDHS 2017–18 employed the 2003 American Heart Association guidelines for hypertension cut-off points [30]. Respondents were classified as hypertensive if their systolic BP over diastolic BP was 140 millimetres of mercury (mmHg)/90mmHg or higher on the survey day. Additionally, individuals who self-reported taking antihypertensive medication were also classified as hypertensive.

### Explanatory variables

We selected two non-modifiable explanatory variables as our greatest interest -age and sex. We categorised age into the following groups: 18–19, 20–24, 25–29, 30–34, 35–39, 40–44, 45–49, 50–54, 55–59, and 60+ years, while respondents' sex categories were male or female. A 5-year age band helps address the heaping in age reporting and can capture any potential nonlinear association with the outcome of interest. Since the hypertension data was collected from adults only, the first age group included respondents aged 18 to 19. Since only 15% of the respondents were aged 60 years or older, we grouped them in one, rather than creating more granular categories with a small number of observations.

We selected the other variables that may influence hypertensive status based on the earlier published literature from Bangladesh [8], India [4], Pakistan [31], and Nepal [5] and on the availability of relevant data on indicators in the BDHS 2017–2018. These included socioeconomic factors-household wealth quintile, educational status, place of residence, and biological factors, namely BMI and fasting blood glucose (FBG) level of both men and women. **Table 1** provides a detailed description of these factors.

**Table 1:** Description of control variables

Factors	Categories	Description
Educational status	No education	No formal education
	Primary incomplete or complete	1–5 years of formal schooling
	Secondary incomplete	6–9 years of formal schooling
	Secondary complete or higher	At least 10 y of formal schooling
Wealth status	Poorest	Household belongs to the lowest wealth quintile
	Poorer	Household belongs to the second-lowest wealth quintile
	Middle	Household belongs to the middle wealth quintile
	Richer	Household belongs to the second-highest wealth quintile
	Richest	Household belongs to the highest wealth quintile
Place of residence	Rural	Resides in rural areas
	Urban	Resides in urban areas
Administrative division	Dhaka	Resides in Dhaka division
	Barisal	Resides in Barisal division
	Chattogram	Resides in Chattogram division
	Khulna	Resides in Khulna division
	Mymensingh	Resides in Mymensingh division
	Rajshahi	Resides in Rajshahi division
	Rangpur	Resides in Rangpur division
	Sylhet	Resides in Sylhet division
BMI	Underweight	BMI is less than 18.5
	Normal	BMI between 18.5 and 24.9
	Overweight/obese	BMI is greater than or equal to 25
Diabetic	Yes	FBG levels ≥7 mmol/L or under medication
	No	FBG levels <7 mmol/L and not under medication
	Not tested	Refused to test diabetes and never tested before

BMI – body mass index, FBG – fasting blood glucose, mmol/L – millimoles per litre

We created the wealth quintile using principal components analysis that considered the quantity and variety of household goods, including items from tables to bicycles and cars, along with housing characteristics such as the source of drinking water, sanitation facilities, and flooring materials [7]. The diagnostic criteria for diabetes adhere to the WHO guidelines of an individual with an FBG level of 7.0 millimoles per litre (mmol/L) or higher to be considered diabetic [32]. The 2017–18 BDHS measured fasting plasma glucose levels by asking participants to refrain from eating or drinking anything other than water for eight hours before testing. The HemoCue Glucose 201 DM system was utilised to test a small blood sample from willing participants. This system converted the FBG readings into fasting plasma glucose values. These results provided a snapshot of the fasting plasma glucose levels within the survey population at the time of the survey. The survey only assessed fasting plasma glucose levels for one day and recorded the results to gain insights into the national impact of this condition.

### Statistical analysis

We performed univariate and bivariate analyses to examine the variation of hypertensive status across sexes, ages, and other sociodemographic groups. We used multivariable logistic regression models to examine the differences in hypertension by sex and age. The outcome of interest was hypertension status among adults, which is binary (hypertensive and normotensive). We designed logistic regression to handle binary outcomes by predicting the probability of an event occurring. It utilises a logistic function (sigmoid curve) to restrict the output between 0 and 1, making it ideal for modelling probabilities. Therefore, we employed multivariable logistic regression, the most widely used regression model in epidemiological studies with binary outcomes. We examined the differences in hypertension by sex for each age group through logit-based marginal probabilities. Additionally, we estimated marginal effects of sex on hypertension for each age group to test the significance of sex differences in hypertension throughout the life course. All analyses were carried out using the appropriate survey design and sampling weights to adjust for the complex survey design characteristics of BDHS 2017–2018. All analyses were conducted using Stata version 15 (College Station, Texas, USA; licensed to icddrb).

### Patient and public involvement

This paper is based on a secondary analysis of publicly available open-source data; therefore, neither patients nor the public were primarily involved in this study.

## RESULTS

### Sociodemographic distribution and prevalence of hypertension

**Table 2** shows the prevalence of hypertension by the background characteristics of the study population – *i.e*. respondents. Approximately 56% of participants were women and 44% were men. The largest age group was 60 or older (15%), followed by the 20–24 and 25–29 years groups (13% each). About three-quarters of the respondents lived in rural areas, with the highest percentage from the Dhaka division (24%), followed by Chattogram (17%) and Rajshahi (14%). The distribution of men and women was similar across the eight divisions; however, slightly more women in Chattogram were hypertensive than men. Aside from educational attainment and BMI, the distribution of other background characteristics was similar between male and female participants. We found that 30% of respondents completed their primary education, and 26% had no education at all. Men had higher levels of educational attainment, with 23% having completed secondary education or higher compared to 15% of women. When we examined BMI, women (30%) had a higher mean BMI than men (18%). Almost a quarter of the respondents were classified as overweight or obese.

**Table 2:** Background characteristics and prevalence of hypertension

Factors	Male		Female		Total		Hypertensive
	n*	**%**	n*	**%**	n*	**%**	**%**
**Total**	-	-	-	-	12476	100.0	27.7
**Sex**							
Men	-	-	-	-	5521	44.3	26.0
Women	-	-	-	-	6955	55.7	29.1
**Age**							
18–19	298	5.4	549	7.9	847	6.8	6.9
20–24	581	10.5	1002	14.4	1583	12.7	9.8
25–29	617	11.2	950	13.7	1567	12.6	13.3
30–34	596	10.8	888	12.8	1485	11.9	19.6
35–39	682	12.4	760	10.9	1442	11.6	27.2
40–44	508	9.2	614	8.8	1122	9.0	30.4
45–49	466	8.4	599	8.6	1065	8.5	37.0
50–54	390	7.1	341	4.9	731	5.9	40.8
55–59	325	5.9	394	5.7	718	5.8	44.8
60+	1057	19.2	859	12.4	1917	15.4	52.1
**Education**							
No education	1282	23.2	1944	27.9	3226	25.9	35.5
Primary	1726	31.3	2016	29.0	3742	30.0	27.3
Secondary incomplete	1239	22.4	1943	27.9	3182	25.5	23.3
Secondary complete or higher	1273	23.1	1053	15.1	2326	18.6	23.6
**Wealth status**							
Poorest	1013	18.4	1362	19.6	2375	19.0	23.9
Poorer	1079	19.5	1358	19.5	2437	19.5	25.4
Middle	1150	20.8	1395	20.1	2545	20.4	27.0
Richer	1113	20.2	1349	19.4	2463	19.7	28.4
Richest	1165	21.1	1491	21.4	2656	21.3	33.4
**Place of residence**							
Urban	1559	28.2	1863	26.8	3422	27.4	28.7
Rural	3962	71.8	5092	73.2	9053	72.6	27.4
**Division**							
Dhaka	1347	24.4	1631	23.5	2978	23.9	23.9
Barisal	304	5.5	386	5.6	690	5.5	33.1
Chattogram	866	15.7	1284	18.5	2149	17.2	30.1
Khulna	698	12.6	836	12.0	1534	12.3	29.9
Mymensingh	455	8.2	569	8.2	1024	8.2	23.4
Rajshahi	793	14.4	980	14.1	1774	14.2	27.6
Rangpur	689	12.5	823	11.8	1513	12.1	31.0
Sylhet	368	6.7	445	6.4	813	6.5	26.4
**BMI**							
Underweight	1093	19.8	1075	15.5	2168	17.4	17.8
Normal	3459	62.7	3828	55.0	7287	58.4	24.4
Overweight and Obese	969	17.5	2052	29.5	3021	24.2	42.9
**Diabetic**							
No	4587	83.1	5918	85.1	10505	84.2	25.8
Yes	537	9.7	636	9.2	1173	9.4	45.8
Not tested	397	7.2	400	5.8	797	6.4	26.0

BMI – body mass index

*Weighted number.

Overall, 28% of Bangladeshi adults aged 18 and older were living with hypertension. The prevalence was slightly higher among women (29%) than men (26%). We observed a steady increase in hypertension in both sexes with advancing age. The prevalence rose from 7% among participants aged 18–19 to 52% among those aged 60 and older. A comparatively higher prevalence was noticed among respondents from higher socioeconomic classes. In contrast, the prevalence was higher among respondents with lower educational levels. The urban-rural differences in hypertension prevalence were minimal. Furthermore, the prevalence increased with a rising BMI. Overweight and obese adults (43%) exhibited a significantly higher prevalence of hypertension, nearly double that of average (24%) or underweight (18%) individuals.

### Sex and age differentials in hypertension across the life course

**Figure 2:**
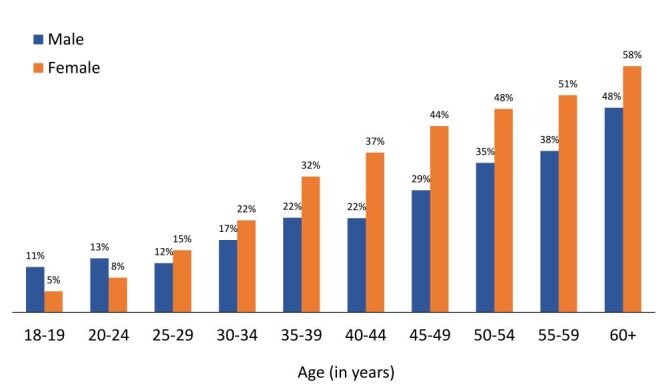
Prevalence of hypertension by sex and age.

**Figure 2** illustrates the age-sex differences in the prevalence of hypertension. Women were less likely to be hypertensive than men until their mid-twenties, with a gradual shift in later years. Among individuals aged 18 to 19, the prevalence of hypertension was six percentage points lower among women than men. However, we saw, among older adults aged 60 and above, the prevalence of hypertension was 10 percentage points higher among women than men. We examined the point at which this transition begins, and our data suggests that more women than men become hypertensive starting in their mid-twenties, with the transition being completed around their mid-thirties **(****Figure 2****)**. During their early twenties, women have a prevalence of hypertension that is five percentage points lower than that of men (**Figure 2**). However, this trend reverses in their early thirties when women have a prevalence of hypertension that is five percentage points higher than that of men. Furthermore, from the age of 25 until their late sixties, the prevalence of hypertension remained higher in women than in their male counterparts.

We conducted additional analysis to definitively assess sex and age differences in the prevalence of hypertension throughout the life course of women and men in Bangladesh. We performed multivariable logistic regression with age-sex interaction, and the models are shown in **Table 3**.

**Table 3:** Association of sex and age with hypertension: results from multivariable logistic regression

Factors	Model I		Model II	
	**AOR**	**95% CI**	**AOR**	**95% CI**
**Sex**				
Male	Reference		Reference	
Female	1.3*	(1.2–1.5)	0.4*	(0.2–0.7)
**Age**				
18–19	Reference		Reference	
20–24	1.3	(0.9–1.9)	1.1	(0.7–1.8)
25–29	1.7*	(1.2–2.3)	1.0	(0.6–1.5)
30–34	2.5*	(1.80–3.45)	1.32	(0.9–2.1)
35–39	4.0*	(2.9–5.5)	1.9*	(1.3–2.9)
40–44	4.8*	(3.5–6.6)	1.9*	(1.3–3.0)
45–49	6.6*	(4.8–9.2)	2.9*	(1.9–4.5)
50–54	9.0*	(6.4–12.8)	3.9*	(2.5–6.1)
55–59	10.8*	(7.8–15.1)	4.6*	(3.0–7.0)
60+	17.1*	(12.5–23.3)	8.0*	(5.4–11.9)
**Age sex interaction (age and sex)**				
Age 18–19, and male	-	-	Reference	
20–24, female	-	-	1.4	(0.7–2.7)
25–29, female	-	-	2.6*	(1.3–5.1)
30–34, female	-	-	2.9*	(1.5–5.6)
35–39, female	-	-	3.6*	(1.9–6.6)
40–44, female	-	-	4.7*	(2.5–8.9)
45–49, female	-	-	4.1*	(2.2–7.8)
50–54, female	-	-	4.4*	(2.2–8.6)
55–59, female	-	-	4.5*	(2.3–8.9)
60+, female	-	-	3.9*	(2.1–7.2)
**Education**				
No education	Reference		Reference	
Primary	1.1	(0.9–1.2)	1.1	(1.0–1.2)
Secondary incomplete	1.1	(1.0–1.3)	1.2†	(1.0–1.4)
Secondary complete or higher	1.1	(0.9–1.3)	1.2	(1.0–1.4)
**Wealth status**				
Poorest	Reference		Reference	
Poorer	1.0	(0.9–1.2)	1.0	(0.9–1.2)
Middle	1.1	(0.9–1.3)	1.1	(0.9–1.3)
Richer	1.2	(1.0–1.4)	1.1	(1.0–1.4)
Richest	1.2*	(1.0–1.5)	1.2	(1.0–1.5)
**Place of residence**				
Urban	Reference		Reference	
Rural	0.9	(0.8–1.1)	0.9	(0.8–1.1)
**Division**				
Dhaka	Reference		Reference	
Barisal	1.7*	(1.4–2.1)	1.7*	(1.3–2.1)
Chattogram	1.4*	(1.2–1.7)	1.4*	(1.2–1.7)
Khulna	1.3*	(1.1–1.6)	1.3*	(1.1–1.6)
Mymensingh	1.1	(0.9–1.4)	1.1	(0.9–1.4)
Rajshahi	1.4*	(1.1–1.7)	1.4*	(1.1–1.7)
Rangpur	1.7*	(1.4–2.1)	1.7*	(1.4–2.1)
Sylhet	1.4*	(1.2–1.8)	1.4*	(1.1–1.8)
**BMI**				
Underweight	Reference		Reference	
Normal	1.8*	(1.6–2.1)	1.8*	(1.6–2.1)
Overweight and obese	4.3*	(3.7–5.1)	4.3*	(3.7–5.1)
**Diabetic**				
No	Reference		Reference	
Yes	1.6*	(1.3–1.8)	1.5*	(1.3–1.8)
Not tested	1.0	(0.8–1.1)	1.0	(0.8–1.2)
Observation used	12680	-	12680	-

AOR – adjusted odds ratio, BMI – body mass index, CI – confidence interval

*P ≤ 0.01.

†*P* < 0.05.

After controlling for the effect of confounders in Model I, sex and age remained significant correlates of hypertension (**Table 3**). The odds of being hypertensive were 1.3 times higher in women than in men. The odds of being hypertensive increased with age. Among all respondents, compared to individuals aged less than 20 years, the odds of being hypertensive increased from 1.7 times (adjusted odds ratio (AOR) = 1.7; 95% CI = 1.2–2.3) in the 25–29 years age group to 17.1 times (AOR = 17.1; 95% CI = 12.5–23.3) among those 60 years or above. Model II, adjusted with age-sex interaction of the women category, shows a significant age-sex interaction in explaining the variation of hypertensive status of women. Estimated adjusted odds ratios (AORs) of interaction terms signify the shift in the burden of hypertension from men to women with increasing age (starting from AOR = 2.6; 95% CI = 1.3–5.1, 25–29-year-old women to AOR = 3.9; 95% CI = 2.1–7.2 for 60+-year-old women). Among all the respondents of this survey, the odds of being hypertensive was 1.9 times higher (AOR = 1.9; 95% CI = 1.3–2.9) in the 35–39 years age group, however, when the age-sex interaction was considered, the odds were much higher (AOR = 3.6; 95% CI = 1.9–6.6) in women of 35–39 years age-group.

**Figure 3:**
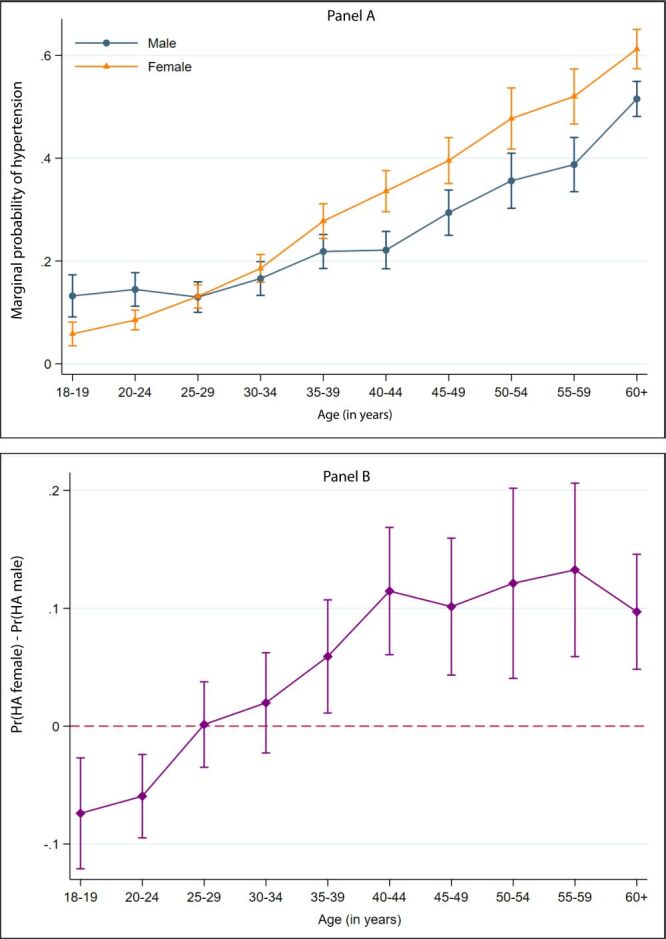
Sex differentials in the marginal probability of being hypertensive: Results from Model II.

We also estimated the adjusted marginal probabilities of being hypertensive in men and women across the age groups using Model II (**Figure 3**). We observed that the trend meets at the 25–29 age category of men and women and then shifts upward for both men and women with a much steeper inclined curve for women. The upper panel of **Figure 3** indicates a sharp increase in the marginal probability of hypertension with increasing age for both men and women. The increasing trend of being hypertensive was steeper among women than in men. The lower panel of **Figure 3** presents the sex differentials in the marginal probabilities of being hypertensive. Before reaching their mid-twenties, the women were significantly less likely to be hypertensive than their male counterparts. The sex differential in the probability of being hypertensive diminished around the mid-twenties. From the early thirties, the probability of hypertension among women was seen to be higher than in men of the same age. Also, around the mid-thirties, women reached a significantly higher probability of hypertension than men. Throughout the rest of their life, women remain more likely to be hypertensive compared to men.

## DISCUSSION

Hypertension is a preventable risk factor for cardiovascular and cerebrovascular morbidities and mortalities [1]. Among the various risk factors for developing hypertension, the influence of sex differences with age remains one of the least explored. This study shows that while it is known that hypertension is more common among men globally, a different scenario is observed in Bangladesh. Women face a significantly higher risk of developing hypertension after they turn 35, and this risk continues to increase with age.

It is evident that the prevalence of hypertension is increasing among Bangladeshi adults. Between the BDHS 2011 and 2017–18 surveys, the prevalence of hypertension among adults aged 35 years and older has risen sharply – from 32% for women and 19% for men in 2011 to 45% for women and 34% for men in 2017–18 [8]. This calculation estimates that among the 48 million women in Bangladesh, 9.7 million suffer from hypertension [7]. This astonishing number of women with hypertension is a serious concern, as maternal hypertension frequently leads to challenging childbirth complications, including severe issues like hemorrhage, preeclampsia, and eclampsia. Furthermore, this presents a significant risk to fetal health and well-being. In Bangladesh, one in five maternal deaths result from indirect causes, with the majority (41%) attributed to pre-conception hypertension, stroke, and associated complications, and in both cases, hypertension serves as a precursor [33].

As we further investigate the differences in the prevalence of hypertension among men and women by age, we find that women in the older age group have a higher likelihood of being hypertensive than men of the same age. Around the ages of 25 to 29, we observed a shift in the trend regarding women being hypertensive. The probability of men being hypertensive begins to decline, while the likelihood of women being hypertensive increases relative to men. When we examined this transition more closely, our adjusted age-sex interaction model revealed that, overall, women had lower odds (AOR = 0.4; 95% CI = 0.2–0.7)) of being affected by hypertension compared to men. Nevertheless, the sex difference in the likelihood of having hypertension, as illustrated in Model II, shows that women start to experience hypertension at a significantly higher rate than men from their early thirties onward.

The authors delve into the reasons behind this shift, which is discussed below.

### Overweight/obesity: women *vs*. men

The mean BMI in Bangladesh is increasing, especially among women, with a rise from 12% in 2007 to 32% in 2017–2018 [34]. A higher BMI is linked to an increased risk of developing hypertension. Previous studies have shown that South Asian populations exhibit a stronger correlation between BMI and hypertension – even at lower thresholds for overweight and obesity – than white European populations [10, 13]. Bangladeshi women are more likely to be overweight or obese compared to women in neighboring countries, exhibiting a higher overall percentage [4, 5]. The most significant differences are seen in the 20 to 29 age group, with 27% of women in Bangladesh classified as overweight or obese, compared to 17% in Nepal and 14% in India [4, 5, 7]. In addition to low-labour-intensive work and reduced physical activity, early childbearing resulting in pregnancy-related weight gain may be a notable area of concern.

Previous studies indicate that early childbearing (before age 20) is prevalent in Bangladesh and correlates with various negative health outcomes. A woman's age at her first pregnancy might also influence the prevalence of hypertension among women in Bangladesh. The mean age for first pregnancy in Bangladesh is 15 years, with nearly 78% of women giving first birth before age 20 [35]. Pregnancy is recognised as a risk factor for hypertension in women who experience pregnancy-induced hypertension [22,36]. Additionally, weight gain during pregnancy and postpartum complications is a common problem among young women in Bangladesh and has been linked to several health issues, including hypertension, gestational diabetes, and pre-eclampsia. Moreover, weight gain during this period is associated with an increased risk of obesity and long-term health complications [37,38].

### Use of combined oral contraceptives

The use of oral contraceptive pills (OCP) has also been associated with a higher likelihood of women developing hypertension [39]. Combined oral contraceptive pills are the most common form of modern contraception used by Bangladeshi women, with 25% of married women in the 15–49 age group using COC pills as a contraceptive method [7]. This contrasts with neighboring countries such as Nepal and India, where the usage of OCPs is relatively low, at 4% in Nepal and 5% in India [40,41]. The percentage of married women using COC pills in Bangladesh is highest among the 30–34 age group (30.4%); however, the rates are similar for women aged 15–39, while the use of OCP among women aged 40 and older is significantly lower [7]. Several studies have associated the use of OCP with temporary increases in BP in women [42,43]. One study shows that married women using COC in Bangladesh have a higher prevalence of hypertension compared to those who use other family planning methods or do not use any method [44].

### Mental disorder throughout the life course: women *vs*. men

The early onset of hypertension in Bangladeshi women may also be linked to stress, anxiety, and depression. Women may encounter more stressful situations than men because of the patriarchal social structure, lack of women’s empowerment, early marriage, conception and childbearing, intimate partner violence, and so on [45]. A lack of facilities and motivation for physical activities, exercise, and freedom of movement, along with other forms of social oppression against women, may also contribute to anxiety and depression [46]. The 2019 National Mental Health Survey reveals that among individuals aged 18 to 99 years, a greater percentage of women (22%) experience various mental disorders compared to men (16%). In Bangladesh, women of all age groups, including those of reproductive age, show a higher prevalence of mental disorders than men, regardless of age group [47]. Mental disorders, including anxiety disorders, depressive disorders, and bipolar disorders, are more prevalent among adult women in Bangladesh [47].

Some studies indicate that anxiety and depression may be linked to an increase in blood pressure, while other research demonstrates a temporary rise in blood pressure mediated by sympathetic activation due to stress [48–50]. Although more studies are needed to establish a direct link between stress, anxiety, depression, and hypertension, stress – whether emotional, social, cultural, or occupational – can lead to various illnesses, including hypertension [45].

### Physical activity: women *vs*. men

In Bangladesh, the higher prevalence of hypertension in women compared to men after a certain age may be linked to lower physical activity, among other factors [51]. Due to early marriage and childbearing, physical activity decreases sooner among women. Generally, physical activity levels in Bangladesh are lower than in other LMICs [52,53]. Previous studies conducted in South Asian countries, including Bangladesh, have shown that women are less active than men [20]. One study found that physical activity at work or during commutes is the main source of total physical activity for the study population of neighboring countries. Men reported spending more time working or commuting than women, indicating that women may be significantly less physically active [21]. Additionally, South Asian women encounter obstacles to engaging in regular physical activities due to socio-cultural factors, such as social stigma and insufficient indoor and outdoor facilities for sports and exercise [54].

The most recent recommendation from WHO to maintain a healthy lifestyle is to undertake 150–300 minutes of moderate-intensity, or 75–150 minutes of vigorous-intensity physical activity for all adults every week. Some equivalent combination of moderate-intensity and vigorous-intensity aerobic physical activity could be done as well [55]. Although women in Bangladesh engage in numerous household chores and childcare activities, these may not be enough in intensity and/or in duration to provide optimal health benefits. The lack of recommended daily exercise and physical activities, which are often absent in the routine of Bangladeshi adult women, contributes to major national non-communicable diseases (NCDs) like hypertension and diabetes.

### Exposure to solid cooking fuel-induced smoke

Exposure to household air pollution from solid cooking fuels like wood, charcoal, dry leaves, and dung is linked to a higher risk of hypertension among women in Bangladesh [25]. Studies have shown that women who spend a considerable amount of time cooking near the stove are at a higher risk of exposure. Additionally, cooking with solid fuels contributes to indoor air pollution, which has been associated with various negative health effects, including hypertension and other cardiovascular diseases in women [56, 57].

### Policy implications

The Non-Communicable Disease Control Program of the Directorate General of Health Services under the Ministry of Health and Family Welfare initiated the implementation of an NCD management model in 2018. This model includes identifying at-risk populations over age 40 in the community and provides screening, diagnosis, treatment, and counselling services at the primary health care level, along with effective referral chain management for beneficiaries. One recommendation we have is to lower the age cut-off for screening hypertension in women in Bangladesh, as also indicated in a published policy brief [34]. The policy brief outlines why and how this can be implemented at a national level. Lowering the current age cut-off of 40 years for screening, which is a crucial step in early identification, can lead to earlier diagnosis of hypertension in women and result in a more targeted approach. To alleviate the burden on patients in primary health care facilities, we recommend basic assessments for follow-up and medication refills for patients already diagnosed at community clinics. This is also highlighted in the policy brief with further details, and the government is already piloting it [34]. Another critical recommendation is to enforce screening for hypertension during antenatal and postnatal visits and connect women who test positive to the national NCD management model. This approach could promote early diagnosis of hypertension through regular follow-up visits, which is already included in the NCD management model.

## CONCLUSIONS

After a certain age, women in Bangladesh face a higher risk of developing hypertension compared to their male counterparts. Further exploration through longitudinal studies and national NCD surveillance is needed to gain a better understanding of the trend of hypertension among women in Bangladesh and to identify the factors that contribute to the higher prevalence of hypertension in Bangladeshi women at a younger age. With the increasing prevalence of hypertension, more women of reproductive age are at risk of developing hypertension and experiencing related adverse health effects for both themselves and their children. Identifying the common factors that contribute to women's higher rates of hypertension in Bangladesh after a certain age will enable better intervention designs, modifications to the national NCD management model, and robust strategic planning to target this high-risk group.

### Strengths

This is a novel study, and to our knowledge, no research has examined the possibility that women may be at greater risk of hypertension after a certain age in Bangladesh. It is anticipated that the findings from this study will contribute to national dialogues and policies if pursued effectively. This research will also pave the way for further investigations into women-centric intervention designs in similar contexts. Another advantage of this study is the use of a nationally representative and well-respected survey, alongside the application of robust statistical methods for data analysis.

### Limitations

In this study, we could not explore and analyse some important risk factors commonly associated with hypertension. Data on dietary habits, physical activity to quantify it, and, though less significant for the female population of Bangladesh, alcohol and tobacco consumption, are some unexplored factors because the BDHS does not collect the relevant information. We also could not investigate the relationship between dosage and usage patterns of oral contraceptives in women with hypertension, as the demographic health survey does not gather this data.
